# What Role Does *PDGFA* Gene Polymorphisms Play in Treating Tennis Elbow with PRP? A Prospective Cohort Study

**DOI:** 10.3390/jcm11123504

**Published:** 2022-06-17

**Authors:** Alicja Jarosz, Karol Szyluk, Joanna Iwanicka, Anna Balcerzyk, Tomasz Nowak, Tomasz Iwanicki, Marius Negru, Marcin Kalita, Tomasz Francuz, Wojciech Garczorz, Sylwia Górczyńska-Kosiorz, Wojciech Kania, Paweł Niemiec

**Affiliations:** 1 Department of Biochemistry and Medical Genetics, School of Health Sciences in Katowice, Medical University of Silesia in Katowice, Medyków 18 St., 40-752 Katowice, Poland; jiwanicka@sum.edu.pl (J.I.); abalcerzyk@sum.edu.pl (A.B.); tnowak@sum.edu.pl (T.N.); tiwanicki@sum.edu.pl (T.I.); pniemiec@sum.edu.pl (P.N.); 2 District Hospital of Orthopaedics and Trauma Surgery, Bytomska 62 St., 41-940 Piekary Śląskie, Poland or karol.szyluk@sum.edu.pl (K.S.); marcin.kalita1991@gmail.com (M.K.); 3 Department of Physiotherapy, Faculty of Health Sciences in Katowice, The Medical University of Silesia in Katowice, 40-752 Katowice, Poland; 4 Trauma and Orthopaedics Department, St. Bernard’s Hospital, Harbour Views Rd, Gibraltar GX11 1AA, Gibraltar; mariusnegru@yahoo.com; 5 Department of Biochemistry, School of Medicine in Katowice, Medical University of Silesia in Katowice, Medyków 18 St., 40-752 Katowice, Poland; tfrancuz@sum.edu.pl (T.F.); wojtekg@sum.edu.pl (W.G.); 6 Department of Internal Medicine, Diabetology and Nephrology, School of Medicine with the Division of Dentistry in Zabrze, The Medical University of Silesia in Katowice, 41-800 Zabrze, Poland; skosiorz@sum.edu.pl; 7 Department of Trauma and Orthopedic Surgery, Multidisciplinary Hospital in Jaworzno, Chełmońskiego 28 St., 43-600 Jaworzno, Poland; wojtekkania@poczta.onet.pl

**Keywords:** platelet-rich plasma, tennis elbow, tendinopathy, *PDGFA*, single nucleotide polymorphisms, gene

## Abstract

Background: This study aims to identify genotype variants of the platelet-derived growth factor alpha polypeptide gene (*PDGFA*) that can influence the individual response to the treatment with platelet-rich plasma (PRP) in tennis elbow patients. Methods: We observed a cohort of 107 patients (132 elbows) with tennis elbow who received treatment with PRP. Patients have been followed-up for two years after PRP injection and the effectiveness of the treatment was measured using universal patient-reported outcome measures (PROMs): visual analog scale (VAS), quick version of disabilities of the arm, shoulder and hand score (QDASH), and patient-rated tennis elbow evaluation (PRTEE). PROMs values, and clinical and platelet parameters were compared between genotype variants of the studied polymorphisms (rs1800814, rs2070958 and rs62433334). Results: The A allele carriers (rs1800814) had significantly lower values of VAS (week 12), QDASH, and PRTEE (weeks 8, 12). The T allele carriers (rs2070958) had significantly lower values of VAS (weeks 8, 12), QDASH, and PRTEE (weeks 4–12). Additional forms of therapy (manual and physical) were necessary significantly more often in GG (rs1800814) and CC (rs2070958) homozygotes. Conclusions: The *PDGFA* gene’s polymorphisms influences the effectiveness of PRP therapy in tennis elbow treatment. The effectiveness of PRP is greater in A allele (rs1800814) and T allele (rs2070958) carriers.

## 1. Introduction

Elbow tenderness and pain with resisted wrist extension are common manifestations of lateral epicondylitis, known as the tennis elbow (TE). The more recent name that reflects the etiology of this dysfunction is enthesopathy of the lateral epicondyle of the humerus [1]. Despite many years of research, the gold standard of treatment has not been established. More and more reports prove the usefulness of autologous platelet-rich plasma (PRP) in the treatment of TE [2,3,4,5]. This therapy is used in many fields of medicine, including regenerative and sports medicine [6]. PRP is a source of over-physiological concentrations of growth factors and other signaling molecules that intensify the body’s healing efforts [2,7]. Platelets play a key role in the regulation of processes related to tissue remodeling and their reorganization following injury or inflammatory processes. They belong to the mediators necessary in the initiation of the body’s immune response, regulation of the angiogenesis process, apoptosis, and recruitment of progenitor cells to maintain the integrity of blood vessels [7]. Most of these processes are mediated by platelets and occur during their degranulation when platelet-derived growth factors (PDGFs) are released. PDGFs are a group of multifunctional proteins that were discovered in the 1970s [8]. They are synthesized based on four polypeptide chains, encoded by the *PDGFA*, *PDGFB*, *PDGFC*, and *PDGFD* genes. The *PDGFA* gene is located within chromosome 7 (7p22) [9] and is highly expressed in most human tissues [10]. The data available so far indicate the significant role of PDGFA in reducing inflammation and accelerating the healing of musculoskeletal injuries [11], osteogenic differentiation, and bone tissue fusion [12].

It should be underlined that despite the potential benefits of PRP therapy, there are significant differences in the results of its use in patients with enthesopathy of the lateral epicondyle of the humerus. The different treatment outcomes may be related to the genetic variation expressed by single nucleotide polymorphisms (SNPs) of the gene encoding the PDGFA growth factor. Therefore, we conducted studies to establish whether selected polymorphisms of the *PDGFA* gene could modulate the response to platelet-rich plasma therapy.

## 2. Materials and Methods

### 2.1. Study Design

This prospective cohort study compares the efficacy of PRP therapy among patients with different genotypic variants of the *PDGFA* gene. Three single nucleotide polymorphisms of the *PDGFA* gene were genotyped. The therapeutic effect was analyzed during a two-year follow-up with the use of patient-reported outcome measures (VAS, QDASH, PRTEE). In addition, the influence of SNPs on clinical phenotype, platelet parameters (both in whole blood and in PRP), and the concentration of the PDGF-AB protein were analyzed. The study was conducted per the STROBE and MIBO guidelines.

### 2.2. Patients

The study population was the same as in our previous work [13]. Patients were recruited for the study between November 2018 and November 2019. Follow-up data was collected until November 2021.

**Figure 1:**
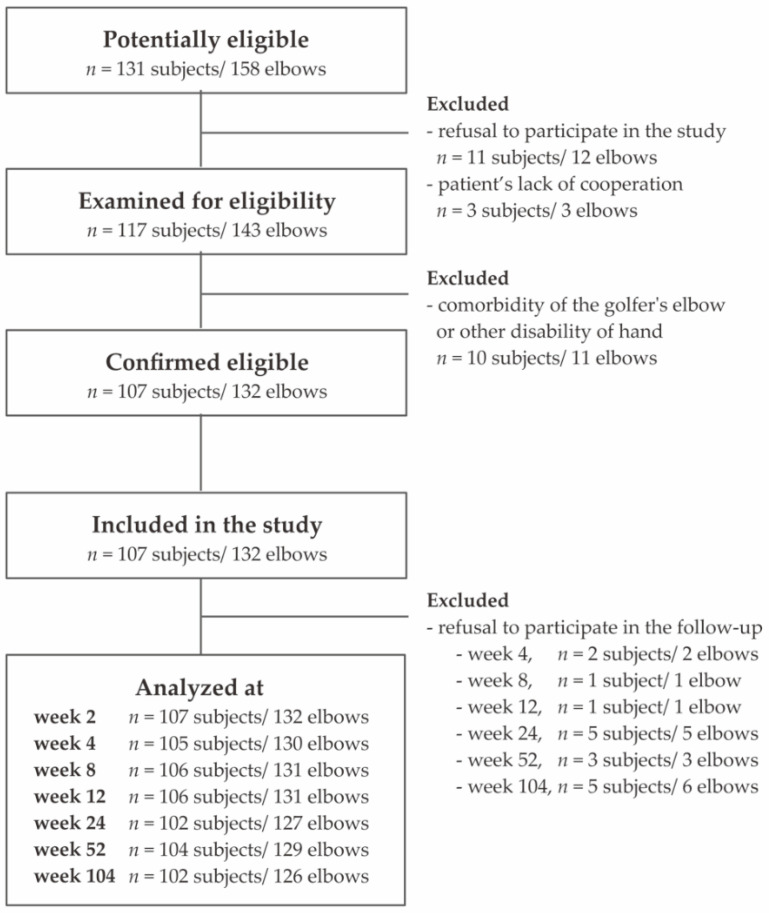
Flowchart of the study selection.

All patients selected in the study were diagnosed with lateral epicondylitis based on patient history and physical examination. The symptoms persisted for at least 3 months before injection and included the following: pain in the region of the common extensor origin radiating distally and proximally, pain and muscle weakness when holding and lifting items, tenderness at palpation over the lateral epicondyle of the humerus, weakening of the grip strength, morning stiffness, repeated activities and/or limb overuse, positive Thomson’s, Mill’s tests, and Cozen’s sign. The exclusion criteria were as follows: rheumatoid arthritis, pregnancy, active malignancy, cervical radiculopathy, cognitive limitations, additional injury/disease of the affected limb, prior surgical intervention, current anti-platelet medication, local steroid injections in the preceding 6 months, and previous PRP injections. Additional post-injection therapy was not an exclusion criterion for ethical reasons. However, the use of physiotherapy, steroids, and non-steroidal anti-inflammatory drugs as well as additional PRP injections were monitored in further follow-up. The flow diagram of the patients included in the study is presented below (Figure 1).

### 2.3. PRP Separation and Injection Procedure

Briefly, PRP was separated from fresh whole blood using the autologous conditioned plasma system (Arthrex GmbH, Munich, Germany). Sodium citrate (MediPac GmbH, Königswinter, Germany) was used as an anticoagulant. Blood was centrifuged using a Rotofix 32A centrifuge (Andreas Hettich GmbH & Co., Tuttlingen, Germany) for 5 min at 1500 rpm and 2.5–3.5 mL of PRP was obtained. Immediately after centrifugation 2.0 to 3.0 mL of PRP was injected in the common extensor origin area. Approximately 0.5 mL was left for protein analyses. The exact procedures of PRP separation and injection were described previously [13].

### 2.4. Whole Blood and PRP Analyses

As in our previous work [13], the complete blood count and the platelet parameters in whole blood and fresh PRP were determined. The PDGF-AB protein concentration was measured in PRP using the Human DuoSet ELISA kit (R&D Systems Inc., Minneapolis, MN, USA).

### 2.5. Follow-Up, Outcomes, Measures of Effectiveness

The effectiveness of PRP therapy was assessed based on a comparison of symptoms between the day of injection (baseline, week 0) and 2, 4, 12, 24, 52, and 104 weeks after administration of PRP.

For assessment of pain and disability, we used VAS, QDASH, and PRTEE questionnaires. The scale for VAS was 0 to 10 and for QDASH and PRTEE it was 0 to 100. Raw values of outcomes (VAS, QDASH, PRTEE) and the differences in outcomes vs. baseline (ΔVAS, ΔQDASH, and ΔPRTEE) were used to determine the effectiveness of therapy.

Minimal clinically important differences (MCIDs) of PROMs were also used as determinants of the effectiveness of the therapy. The therapy was considered effective when the mean difference of PROM (between the follow-up point and the baseline week 0) exceeded the value of 1.5 points for VAS [14], 15.8 points for DASH [15], and 11 points for PRTEE [16].

### 2.6. Genetic Analyses

The procedure used for DNA isolation and genotyping was the same as in our previous study [13]. The MasterPure genomic DNA purification kit (Epicenter Technologies, Madison, WI, USA) was used for DNA isolation. SNPs of the *PDGFA* gene were genotyped using the TaqMan Predesigned SNP Genotyping Assay kits and the 7300 Real-Time PCR System (Thermo Fisher Scientific, Carlsbad, CA, USA). We re-genotyped 10–15% of samples to check the accuracy of genotyping. Repeatability of results reached 100%.

The polymorphisms for the analysis were selected from the Database of SNPs of the National Center for Biotechnology Information, U.S. National Library of Medicine [17]. We chose only SNPs where the frequency of the minor allele was ≥20%.

There were rs1800814 (G > A), rs2070958 (T > C), rs62433334 (C > G) variants. They are all located within the introns of the *PDGFA* gene.

### 2.7. Statistical Analyses

The Statistica 13.0 software (TIBCO Software Inc., Carlsbad, CA, USA) was used for data analysis. Genetic data were analyzed in additive (comparison of genotypes) and recessive/dominant models (analysis of carrier-state). The Hardy–Weinberg equilibrium was tested with the χ^2^ test. Yates’ correction was applied for subgroups of less than ten subjects. Study size and power analysis were calculated using Epi-InfoTM 7.2.1.0 software (Centers for Disease Control and Prevention, Atlanta, GA, USA). The normality of the distribution of quantitative data was assessed using the Shapiro–Wilk test. As the quantitative variables had a distribution other than normal, nonparametric tests were used to compare them (Kruskal–Wallis test in the additive model, Mann–Whitney U test in the recessive/dominant model). All quantitative data were reported as the median and their spread as quartile deviation (QD). Spearman’s rank correlation coefficient (r_s_) was used as a nonparametric measure of correlation. Statistical significance was assumed at *p* < 0.050. For multiple comparisons, *p* values were corrected using the Bonferroni correction.

## 3. Results

### 3.1. General Characteristics of the Study Group

The study cohort consisted of 107 patients with median age (±QD) of 46.00 ± 5.50 years and a median BMI (±QD) of 25.65 ± 2.00. More than half of the study group were women (58.3% vs. 41.7%). As in some patients two elbows were treated, the total number of elbows analyzed was 132 and 65.2% of them concerned the dominant hand. 

The median platelet concentration (10^9^/L) was 240.00 ± 40.50 in the whole blood and 343 ± 65.00 in the PRP. The median PDGF-AB concentration (ng/mL) in the PRP was 8.27 ± 2.40. The most common comorbidities were hypertension (13.6%), thyroid disease (11.4%), and gout (6.1%). There were 22 (16.6%) cigarette smokers in the group. The median (±QD) number of units of alcohol consumed per week was 1.00 ± 1.00. Other demographic and clinical data are presented in Table 1.

### 3.2. Factors Influencing Platelets and PDGF-AB Concentration

Traditional factors that influenced the concentration of platelets in whole blood were sex and hypertension. The median (±QD) concentration of platelets (10^9^/L) was higher in females than in males (261.50 ± 33.00 vs. 224.00 ± 38.75, *p* = 0.000) and in hypertensive patients than in normotensives (285.00 ± 40.00 vs. 230.00 ± 34.25, *p* = 0.029). Platelet concentration in whole blood correlated with platelet and PDGF-AB concentrations in PRP. There was also an inverse correlation between platelet concentration and the number of units of alcohol consumed per week (Table 2). The median (±QD) concentration of platelets (10^9^/L) in PRP was higher in cigarette smokers than in non-smokers (390.50 ± 113.50 vs. 338.00 ± 60.00, *p* = 0.017). Platelet concentration in PRP correlated with the concentration of platelets in whole blood, PDGF-AB level, BMI, and the number of cigarettes smoked daily (Table 2). The median (±QD) concentration of PDGF-AB (ng/mL) was higher in cigarette smokers than in non-smokers (10.43 ± 1.62 vs. 7.76 ± 1.98, *p* = 0.001). PDGF-AB level correlated with the number of cigarettes smoked daily and was inversely correlated with age (Table 2).

Other non-genetic factors did not affect the concentration of platelets and PDGF-AB protein in the present study. The influence of the *PDGFA* gene polymorphisms on platelet parameters was presented separately, in Section 3.5.

### 3.3. Factors Influencing PROMs Values

Among the many analyzed parameters (sex, age, BMI, the presence of comorbidities, additional forms of therapy, cigarette smoking, alcohol consumption), statistically significant differences (*p* < 0.050) were demonstrated between women and men in VAS (weeks 0, 24), QDASH (weeks 0–104), and PRTEE (weeks 0, 2, 8–104) values (Table S1). As these differences were present at baseline in each of the PROMs, with no significant differences in the values of ΔVAS, ΔQDASH, and ΔPRTEE, sex cannot be considered a factor significantly influencing the effectiveness of therapy in the present study. Physical therapy reported during follow-up was related to poorer PRP therapy effectiveness at weeks 8–24 and 102 (significantly higher VAS, QDASH, and PRTEE values, *p* < 0.050). 

Other non-genetic factors did not affect the values of PROMs. The influence of the *PDGFA* gene polymorphisms on PROMs values was presented separately, in Section 3.6.

### 3.4. PDGFA Gene Polymorphisms and Patients Characteristics

Genotyping data were obtained for all subjects (132 elbows). Genotype frequencies of each of the analyzed SNPs were compatible with the Hardy–Weinberg equilibrium (*p* > 0.050). The frequencies of genotypes and alleles of the analyzed polymorphisms are presented in Table 3.

The frequencies and medians of basic demographic and clinical characteristics of patients were compared between the different genotypes of the polymorphisms studied. The analysis included those parameters that could influence the treatment efficacy and those that were related or correlated with platelet parameters in Section 3.2 (Table S2). 

In the additive model, a higher frequency of cigarette smokers among AA homozygotes than AG heterozygotes of the rs1800814 polymorphism was observed (29.73% vs. 10.00%, *p* = 0.028). In GG homozygotes of this polymorphism, physical therapy was also used more often during follow-up than in AG heterozygotes (57.14% vs. 31.67%, *p* = 0.015). This relationship was also present in the recessive/dominant model (GG vs. AA + AG, *p* = 0.040).

In the case of the rs2070958 polymorphism, a greater proportion of men was demonstrated in patients with the CT genotype relative to TT (53.57% vs. 32.08%, *p* = 0.024). Manual therapy during follow-up was more common in CC homozygotes than in CT heterozygotes (60.87% vs. 35.71%, *p* = 0.019) as well as TT homozygotes (60.87% vs. 20.75%, *p* = 0.000). Also, in this case, the differences were statistically significant in the recessive/dominant model (CC vs. CT + TT, *p* = 0.001). 

In GG homozygotes of the rs62433334 polymorphism, physical therapy was used more often during follow-up, in relation to C allele carriers (80.00% vs. 39.34%, *p* = 0.030).

### 3.5. PDGFA Gene Polymorphisms and Platelet Parameters

In the additive model, the GG homozygotes of the rs1800814 polymorphism were characterized by a higher concentration of PDGF-AB than the AG heterozygotes (*p* = 0.017). Details are provided in Table S3. 

In the recessive/dominant model, PDGF-AB concentration was shown to be higher in GG homozygotes (rs1800814) relative to A allele carriers (*p* = 0.019). PDGF-AB concentration was also higher in CC homozygotes (rs2070958) than in T allele carriers (*p* = 0.047). Detailed data are presented in Table S4.

### 3.6. PDGFA Gene Polymorphisms and PROMs

In the additive model (Table 4 and Table S5), patients with AG genotype of the rs1800814 polymorphism had a significantly better response to PRP therapy in weeks 8 (higher ΔQDASH) and 12 (lower VAS) than GG homozygotes. Furthermore, AA homozygotes had lower values of QDASH (at week 8) than GG homozygotes (Table 4). There were not statistically significant differences in PROMs values between AA and AG genotypes. The CC homozygotes of the rs2070958 polymorphism had worse therapeutic effects than: (1) CT heterozygotes at weeks 8 (higher values of QDASH) and 12 (higher values of QDASH, VAS, and PRTEE, lower values of ΔQDASH); (2) TT homozygotes at weeks 4 (lower values of ΔQDASH), 8 (higher values of QDASH and PRTEE, lower values of ΔQDASH) and 12 (higher values of QDASH, VAS, and PRTEE, lower values of ΔQDASH). The GG homozygotes of the rs62433334 polymorphism had a worse therapeutic effect than CG heterozygotes at week 104 (lower values of ΔQDASH and ΔPRTEE).

**Figure 2:**
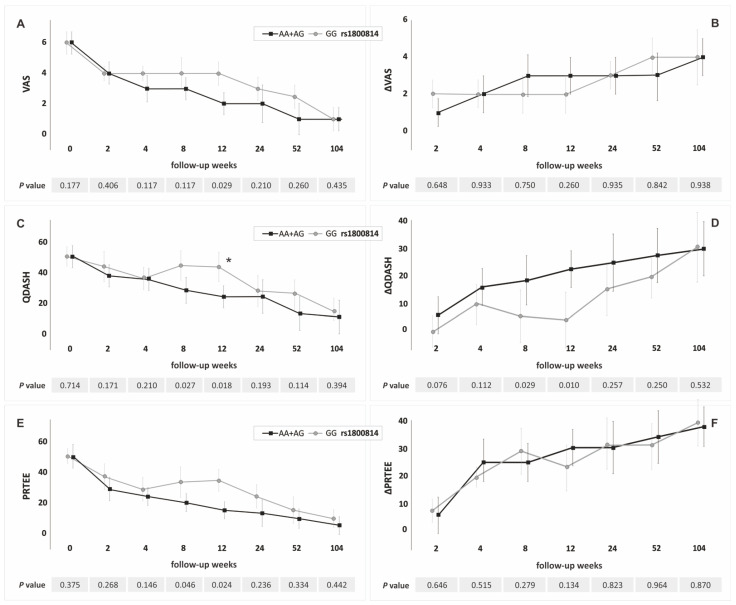
Medians (±QD) of PROMs values in respect to genotype variants of the *PDGFA* gene rs1800814 polymorphism (recessive/dominant model): (**A**) for VAS; (**B**) for ΔVAS; (**C**) for QDASH; (**D**) for ΔQDASH; (**E**) for PRTEE; (**F**) for ΔPRTEE. * Statistically important differences (*p* < 0.050) in the percentage of patients who exceeded minimally clinically important difference (MCID). Legend: QD, quartile deviation; PROM, patient-reported outcome measure; VAS, visual analog scale; QDASH, quick version of disabilities of the arm, shoulder and hand score; PRTEE, patient-rated tennis elbow evaluation.

**Figure 3:**
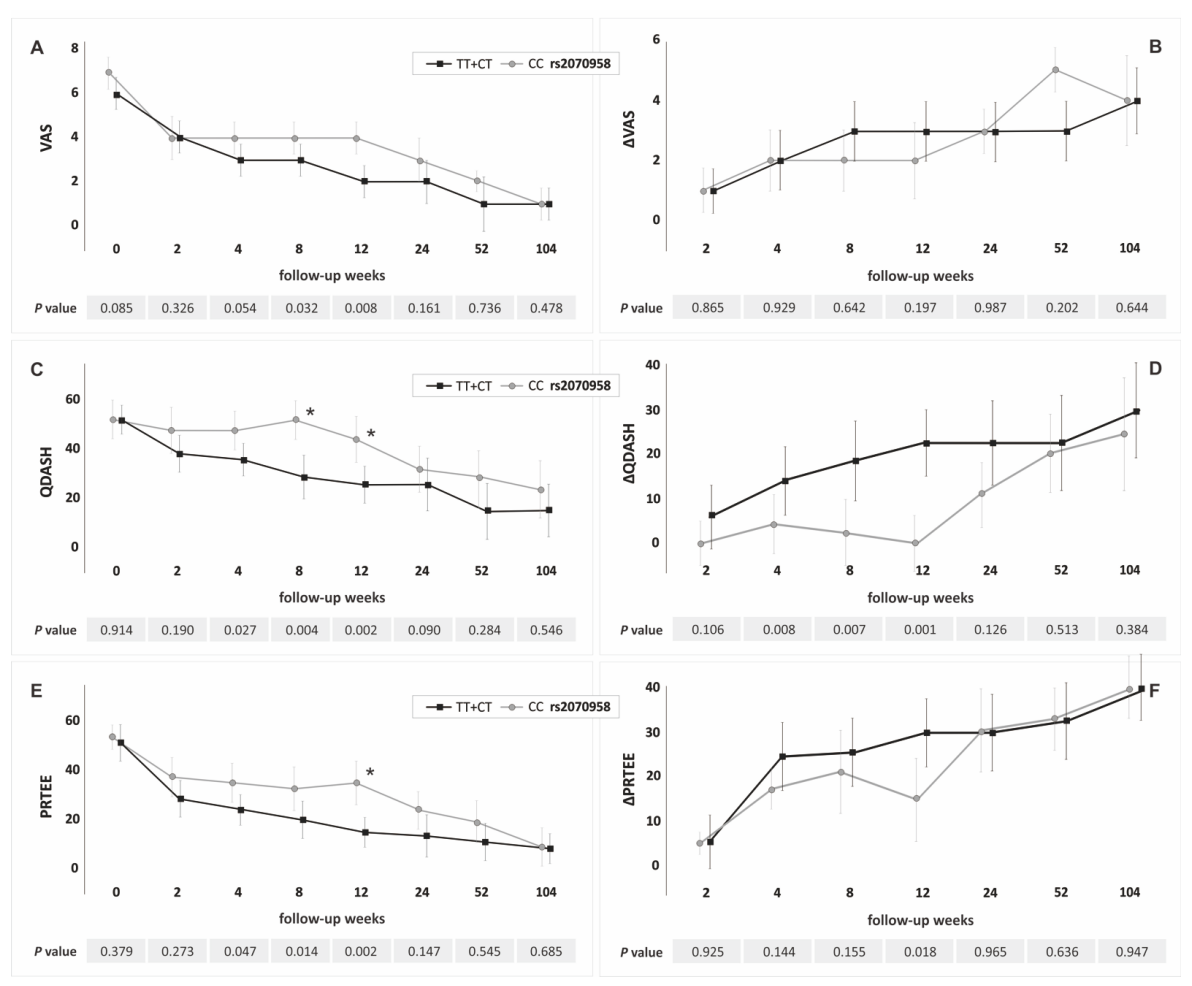
Medians (±QD) of PROMs values in respect to genotype variants of the *PDGFA* gene rs2070958 polymorphism (recessive/dominant model): (**A**) for VAS; (**B**) for ΔVAS; (**C**) for QDASH; (**D**) for ΔQDASH; (**E**) for PRTEE; (**F**) for ΔPRTEE. * Statistically important differences (*p* < 0.050) in the percentage of patients who exceeded minimally clinically important difference (MCID). Legend: QD, quartile deviation; PROM, patient-reported outcome measure; VAS, visual analog scale; QDASH, quick version of disabilities of the arm, shoulder and hand score; PRTEE, patient-rated tennis elbow evaluation.

In the recessive/dominant model the A allele carriers (rs1800814), compared to GG homozygotes, had significantly lower values of VAS at week 12, QDASH, and PRTEE at weeks 8, 12 and significantly higher values of ΔQDASH at weeks 8, 12 (Figure 2, Table S6). The T allele carriers (rs2070958) had significantly lower values of VAS in weeks 8, 12, QDASH and PRTEE at weeks 4–12, and significantly higher values of ΔQDASH at weeks 4–12 than CC homozygotes (Figure 3, Table S7). The GG homozygotes of the rs62433334 polymorphism had lower values of ΔQDASH and ΔPRTEE at week 104 than C allele carriers (Table S8). 

The percentage of patients who exceeded the minimal clinically significant difference for QDASH and PRTEE differentiated the genotypes of the rs1800814 and rs2070958 polymorphisms (Figure 2 and Figure 3). Carriers of the A allele (rs1800814) and carriers of the T allele (rs2070958) more frequently exceeded the MCID than the GG and CC homozygotes.

## 4. Discussion

Our study indicates that the effectiveness of PRP in tennis elbow is much higher in carriers of the A allele (rs1800814) and carriers of the T allele (rs2070958) of the *PDGFA* gene. The noted effect is short-term and concerns observations in weeks 4–12 of follow-up, depending on the genetic variant, adopted inheritance model, and analyzed PROM. In C allele carriers of the rs62433334 polymorphism, the therapy is beneficial only in the long-term perspective (higher values of ΔQDASH and ΔQDASH at week 104 of follow-up than in GG homozygotes).

One of the main problems in interpreting the results of genetic studies, especially those conducted in virgin areas for genetics, is the lack of reference studies. The only study that may indirectly explain the observations of the present study is the work of Zeckey et al., regarding the role of variants of cytokine genes in the regeneration processes [18]. Out of twenty polymorphisms analyzed, there were two SNPs of the *PDGFA* gene (rs1800814 and rs62433334), which were also studied in our present research. It marked the GGC haplotype (within the polymorphisms rs1800814, rs62433334, and rs13309625 of the *PDGFA* gene), which determined the lack of proper aseptic union of the long bones of the lower limbs more than three times (OR = 3.57; 95% CI: 1.21–10.52; *p* = 0.020). According to the authors of the study, polymorphisms within the *PDGFA* gene seem to be a genetic risk factor for the development of non-unions of the lower extremity following a fracture [18]. These results are consistent with those obtained in the present work. Also in our research, the presence of the G allele (precisely GG homozygosity) in each of the polymorphisms was associated with a lower efficiency of the therapy. It should also be noted that in our study, additional physical therapy treatment during follow-up was more frequently necessary in GG homozygotes of both polymorphisms than in A allele carriers (rs1800814) and C allele carriers (rs62433334). A similar relationship was also observed in the case of the rs2070958 polymorphism, where the higher frequency of manual therapy during follow-up was for CC homozygotes relative to T allele carriers.

We were somewhat surprised that both the A allele carriers (rs1800814) and the T allele carriers (rs2070958) had a lower concentration of PDGF-AB than the GG and CC homozygotes of the studied polymorphisms, i.e., patients with worse therapeutic effectiveness. It was even more surprising that the factors that were related to/correlated with the concentration of PDGF-AB in the current study (platelet concentration, age, cigarette smoking) did not differentiate the variants of the studied polymorphisms in the recessive/dominant model. Only in the additive model, among the AA homozygotes of the rs1800814 polymorphism, a higher proportion of cigarette smokers was observed than in the AG heterozygotes. However, subjects of both genotypes belong to the A allele carriers. Moreover, there are no differences between them in PROMs and platelet parameters, including PDGF-AB concentration.

PDGF is a strong mitogenic factor involved in musculoskeletal tissue regeneration [19,20,21,22,23,24]. The results of in vitro studies suggest that the mitogenic response to PDGF-AB may be dependent on both the time of exposure and the dose of the cytokine. Manoranjan et al. analyzed the effect of PDGF-AB on human periodontal ligaments fibroblasts (hPDLF) at 50, 100, and 150 ng/mL dosages at 24, 48, and 72 h of time duration [25]. This study showed that PDGF-AB induced a maximal mitogenic response in hPDLF within 48 h and an intermediate dose of 100 ng/mL, while at the dose of 150 ng/mL a decrease in the mitotic potential of PDGF-AB was observed [25]. Significant methodological discrepancies, as well as the research model used, make it difficult to relate the results of this work to the results of the current study. It seems obvious, however, that the dynamics of PDGF-AB-dependent processes will be different in the in vitro and in vivo systems. The concentration of PDGF-AB in a PRP preparation will not be the only determinant of therapeutic success in the in vivo system. It will also depend on the sex and age of the patient [26,27], the general condition of the body and physical activity [28], but also the use of medications, e.g., nonsteroidal anti-inflammatory drugs [29], the presence of comorbidities, and finally the cellular composition of PRP [30]. In our study, however, we did not find significant differences in clinical and biochemical parameters between patients with particular variants of the *PDGFA* gene, which would explain the observed differences in PDGF-AB concentration. Returning to the studies documenting the influence of cytokine concentration on the effectiveness of therapy, the analysis of the relationship between the PRP composition and the effectiveness of PRP treatment of tennis elbow was carried out by Lim et al. [31]. The results of this study indicated that parameters such as the concentration of platelets, PDGF-AB, PDGF-BB, vascular endothelial growth factor (VEGF), epidermal growth factor (EGF), and interleukin 1β (IL-1β) were not correlated with the progress of PRP treatment, and those that correlated with them were the concentration of white blood cells and transforming growth factor β (TGF-β) [31]. In addition, Sundman et al. showed that the injection of PRP leads to an increase in the concentration of PDGF-AB, TGF-β, and IL-1β, moreover the level of increase depends on the cellular composition of PRP [30]. Moreover, de Melo Viveiros et al. observed that treatment with PRP causes a significant increase in the production of interleukin 6 (IL-6) and interleukin 8 (IL-8) in lateral epicondylitis derived cells [32]. It is unknown how these cytokines affect the regeneration process during tennis elbow. Due to the limited knowledge on this subject, further research is necessary which would focus on determining the influence of growth factors on the effectiveness of PRP therapy in the treatment of tendinopathies. Such an analysis will not be complete if it does not take into account the basic demographic, biochemical, and clinical factors which, on the one hand, may affect the composition of PRP and, on the other hand, modify the body’s response to the therapy.

An analysis of the literature on the genetics of musculoskeletal system injuries also reveals large gaps in knowledge, especially in terms of prognostic factors, potentially modifying the body’s response to platelet-rich plasma therapy. Such factors certainly include variants of the cytokine genes present in PRP. In our previous work, we showed that specific genotypes of the *PDGFB* gene, namely TT (rs2285099), CC (rs2285097), and AA (rs2247128) were associated with a significantly higher effectiveness of PRP therapy. during almost the entire one-year follow-up period [13]. These data, together with the results of the present study, suggest that the body’s response to the PRP therapy may be the result of the interaction of multiple gene variants (multi-gene inheritance model). A review of the contemporary literature shows that the goal of most of the remaining studies was to look for genetic risk factors for tendinopathy. The vast majority of these works are case-control studies. Due to the applied research model, their results are characterized by high variability resulting from different criteria for selecting the study and control groups, as well as ethnic differences. In the light of current knowledge, well-established factors of susceptibility to tendinopathy are the polymorphic variants of collagen, type I, alpha-1 (*COL1A1*) [33], collagen, type V, alpha-1 (*COL5A1*) [34] and matrix metalloproteinase 3 (*MMP3*) [35] genes. However, little is known about their effect on the prognosis of tennis elbow therapy, as such studies have not been conducted so far.

Our study was limited by the relatively small size of the study group and the lack of formal protocol for post-injection physiotherapy. The size of the group is a strong limiting factor in the analysis of qualitative variables, whereas the main data analyzed in the current study were quantitative variables (PROMs, platelet parameters) for which the number of patients was sufficient. Regarding the rehabilitation protocol, we considered it unethical to influence patients’ therapeutic decisions. As a result, we obtained additional data and identified genotypes that required physiotherapeutic intervention during follow-up more frequently. Our results are not only a statement or not of a relationship between the *PDGFA* gene polymorphism and the effectiveness of tennis elbow treatment. In our opinion, the most important thing about them is that they combine clinical aspects and biological issues not in a purely academic but also in a practical form. Perhaps one day it will be enough to test the type of polymorphism before applying PRP therapy and implement it in patients susceptible to treatment.

In summary, the results of our study show that the influence of polymorphism of genes encoding platelet growth factors may be related to specific results of tennis elbow treatment. In addition, according to the authors, the perspective of examining the effectiveness of PRP treatment in other musculoskeletal conditions in terms of gene polymorphism should be used more widely.

## 5. Conclusions

In conclusion, polymorphic variants of the *PDGFA* gene modify the effectiveness of platelet-rich plasma therapy in tennis elbow patients. The carrier-state of the A (rs1800814) and T (rs2070958) alleles of the *PDGFA* gene predispose to a better response to PRP therapy. Therapy is the least effective in the GG homozygotes of both polymorphisms. After PRP injection, GG homozygotes more often require additional forms of therapy (physical therapy, manual therapy).

The *PDGFA* gene polymorphisms may find clinical application in the future as genetic markers of the efficacy of PRP therapy in the treatment of tennis elbow. We hope that a personalized approach to treating musculoskeletal injuries will improve patient satisfaction, increase quality, and reduce treatment costs, which should be the overriding goal of genetic research in each of the fields of medicine.

## Figures and Tables

**Table 1:** Characteristics of the study group before the PRP injection.

Characteristics			
Subjects	Number of subjects, *n* (%)	107	(100.0)
	Single tennis elbow, *n* (%)	82	(76.6)
	Bilateral tennis elbow, *n* (%)	25	(23.4)
Elbows	Number of elbows, *n* (%)	132	(100.0)
	Tennis elbow in the dominant hand, *n* (%)	86	(65.2)
	Tennis elbow in the non-dominant hand, *n* (%)	46	(34.8)
Demographics	Females, *n* (%)	77	(58.3)
	Age, median ± QD	46.00	5.50
BMI	BMI, median ± QD	25.65	2.00
	BMI ≥ 25, *n* (%)	86	(65.2)
Cigarette smoking	Current smoking, *n* (%)	22	(16.6)
	Former smoking, *n* (%)	48	(36.4)
Main comorbidities	Diabetes mellitus, *n* (%)	4	(3.0)
	Gout, *n* (%)	8	(6.1)
	Thyroid diseases, *n* (%)	15	(11.4)
	Hypercholesterolemia, *n* (%)	7	(5.3)
	Hypertension, *n* (%)	18	(13.6)

**Table 2:** Spearman’s rank correlation coefficients (r_s_) between factors influencing platelets and PDGF-AB concentrations.

Variables	PLT WB, 10^9^/L	PLT PRP, 10^9^/L	PDGF-AB, ng/mL	Age	BMI	Cigarettes/Day	Alcohol Units/Week
PLT WB, 10^9^/L	-	0.57 *	0.45 *	−0.08	0.10	0.11	−0.31 *
PLT PRP, 10^9^/L	0.57 *	-	0.71 *	−0.12	0.32 *	0.19 *	−0.04
PDGF-AB, ng/mL	0.45 *	0.71 *	-	−0.23 *	0.16	0.26 *	−0.05
Age	−0.08	−0,12	−0.23 *	-	0.20 *	−0.05	−0.06
BMI	0.10	0.32 *	0.16	0.20 *	-	0.00	0.10
Cigarettes/day	0.11	0.19 *	0.26 *	−0.05	0.00	-	−0.03
Alcohol units/week	−0.31 *	−0.04	−0.05	−0.06	0.10	−0.03	-

* *p* < 0.050. Legend: BMI, body mass index; PDGFA, platelet-derived growth factor-alpha; PLT, platelets; PRP, platelet-rich plasma; WB, whole blood.

**Table 3:** Frequency of genotypes and alleles of analyzed SNPs of the *PDGFA* gene.

SNP	Genotypes	*n* (%)	Alleles	*n* (%)
rs1800814	AA	37 (28.0)	A	134 (50.8)
	AG	60 (45.5)	G	130 (49.2)
	GG	35 (26.5)		
	AA + AG	97 (73.5)		
	GG + AG	95 (72.0)		
rs2070958	CC	23 (17.4)	C	102 (38.6)
	CT	56 (42.4)	T	162 (61.4)
	TT	53 (40.2)		
	CC + CT	79 (59.8)		
	TT + CT	109 (82.6)		
rs62433334	CC	65 (49.2)	C	187 (70.8)
	CG	57 (43.2)	G	77 (29.2)
	GG	10 (7.6)		
	CC + CG	122 (92.4)		
	GG + CG	67 (50.8)		

**Table 4:** Median (±QD) values of PROMs for genotypes of the *PDGFA* gene polymorphisms (additive model).

PROM	Week	Median ± QD in Respective Genotypes			*p*-Value			
		rs1800814			Kruskal–Wallis	AA vs. AG	AA vs. GG	AG vs. GG
		AA	AG	GG				
QDASH	8	25.00 ± 18.18	29.55 ± 15.34	45.45 ± 18.18	0.049	0.892	0.047	0.294
ΔQDASH	8	18.18 ± 19.87	22.73 ± 14.38	4.54 ± 20.46	0.035	1.000	0.125	0.040
VAS	12	3.00 ± 1.50	2.00 ± 1.50	4.00 ± 1.50	0.039	0.582	0.816	0.038
			rs2070958		Kruskal–Wallis	CC vs. CT	CC vs. TT	CT vs. TT
		CC	CT	TT				
ΔQDASH	4	4.54 ± 13.63	14.77 ± 15.34	15.90 ± 18.18	0.017	0.123	0.013	0.836
QDASH	8	52.27 ± 15.91	29.55 ± 14.77	29.54 ± 20.45	0.014	0.026	0.018	1.000
ΔQDASH	8	2.27 ± 14.77	17.04 ± 16.60	18.18 ± 18.18	0.012	0.118	0.009	0.677
PRTEE	8	33.00 ± 17.25	21.75 ± 16	19.50 ± 13.50	0.042	0.105	0.043	1.000
VAS	12	4.00 ± 1.50	2.00 ± 1.50	2.00 ± 1.50	0.028	0.030	0.071	1.000
QDASH	12	43.18 ± 18.18	27.27 ± 13.07	25.00 ± 20.45	0.008	0.008	0.020	1.000
ΔQDASH	12	0.00 ± 12.50	22.72 ± 14.21	23.64 ± 19.20	0.004	0.013	0.004	1.000
PRTEE	12	36.50 ± 17.50	15.75 ± 11.50	18.00 ± 13.25	0.007	0.007	0.017	1.000
			rs62433334		Kruskal–Wallis	CC vs. CG	CC vs. GG	CG vs. GG
		CC	CG	GG				
ΔQDASH	104	34.09 ± 21.59	27.27 ± 22.27	52.27 ± 9.09	0.031	0.295	0.369	0.044
ΔPRTEE	104	38.50 ± 15.75	33.50 ± 15.50	55.25 ± 11.25	0.035	0.650	0.189	0.034

Legend: QD, quartile deviation; PROM, patient-reported outcome measure; VAS, visual analog scale; QDASH, quick version of disabilities of the arm, shoulder and hand score; PRTEE, patient-rated tennis elbow evaluation.

## Data Availability

Not applicable.

## Supplementary Materials

The following supporting information can be downloaded at: https://www.mdpi.com/article/10.3390/jcm11123504/s1, Table S1: PROMs values in males and females; Table S2: The frequencies (%) and medians (±QD) of basic demographic and clinical characteristics of patients in relation to genotypes of the *PDGFA* gene polymorphisms; Table S3: Whole blood (WB) and platelet-rich plasma (PRP) parameters values in individuals with particular genotypes of the *PDGFA* gene polymorphisms (additive model); Table S4: Whole blood (WB) and platelet-rich plasma (PRP) parameters values in individuals with particular variants of the *PDGFA* gene polymorphisms (rs1800814, rs2070958 and rs62433334) in recessive/dominant model; Table S5: PROMs values in individuals with particular genotypes of the *PDGFA* gene polymorphisms (rs1800814, rs2070958 and rs62433334); Table S6: Platelet parameters and PROMs values in GG homozygotes and A allele carriers of the rs1800814 *PDGFA* gene polymorphism; Table S7: Platelet parameters and PROMs values in CC homozygotes and T allele carriers of the rs2070958 *PDGFA* gene polymorphism; Table S8: Platelet parameters and PROMs values in GG homozygotes and C allele carriers of the rs62433334 *PDGFA* gene polymorphism.
