# Supplementary material for: What Role Does PDGFA Gene Polymorphisms Play in Treating Tennis Elbow with PRP? A Prospective Cohort Study
Source: J Clin Med. 2022 Jun 17;11(12):3504. doi: 10.3390/jcm11123504 (PMC9224593; doi:10.3390/jcm11123504)
Supplement: Supplementary file 1 [file jcm-11-03504-s001.zip › Table S1.pdf]

**Table S1.** PROMs values in males and females.

| Parameter | | males | | females | | p Mann-Whitney U test |
|---|---|---|---|---|---|---|
| | week | median | ±QD | median | ±QD | |
| VAS | 0 | 5.00 | 1.75 | 6.00 | 2.00 | **0.032** |
| | 2 | 3.00 | 1.50 | 4.00 | 1.50 | 0.423 |
| | 4 | 3.00 | 1.00 | 4.00 | 1.50 | 0.068 |
| | 8 | 2.50 | 2.00 | 3.50 | 2.00 | 0.220 |
| | 12 | 2.00 | 1.50 | 3.00 | 2.00 | 0.356 |
| | 24 | 1.00 | 2.00 | 3.00 | 2.00 | **0.013** |
| | 52 | 1.00 | 2.00 | 2.00 | 2.25 | 0.219 |
| | 104 | 0.00 | 1.00 | 1.00 | 1.50 | 0.143 |
| ΔVAS (vs week 0) | 2 | 1.00 | 1.00 | 2.00 | 2.00 | 0.054 |
| | 4 | 2.00 | 2.00 | 2.00 | 2.00 | 0.549 |
| | 8 | 2.00 | 2.00 | 3.00 | 2.00 | 0.351 |
| | 12 | 2.00 | 2.50 | 3.00 | 2.00 | 0.280 |
| | 24 | 2.00 | 2.00 | 3.00 | 2.00 | 0.785 |
| | 52 | 2.00 | 2.00 | 4.00 | 2.50 | 0.446 |
| | 104 | 3.00 | 2.25 | 4.00 | 2.00 | 0.411 |
| QDASH | 0 | 42.05 | 13.66 | 59.09 | 12.48 | **0.000** |
| | 2 | 29.55 | 13.07 | 45.45 | 15.91 | **0.003** |
| | 4 | 30.68 | 11.93 | 43.18 | 14.77 | **0.005** |
| | 8 | 29.55 | 14.20 | 36.36 | 19.32 | **0.017** |
| | 12 | 25.00 | 14.77 | 32.95 | 20.45 | **0.016** |
| | 24 | 13.64 | 15.91 | 34.09 | 21.02 | **0.000** |
| | 52 | 13.64 | 16.48 | 27.27 | 22.16 | **0.007** |
| | 104 | 2.27 | 11.36 | 19.09 | 21.59 | **0.009** |
| ΔQDASH (vs week 0) | 2 | 4.55 | 10.23 | 6.82 | 14.77 | 0.384 |
| | 4 | 7.16 | 15.40 | 15.90 | 14.14 | 0.074 |
| | 8 | 9.09 | 18.75 | 15.91 | 17.05 | 0.196 |
| | 12 | 14.77 | 16.29 | 20.45 | 15.91 | 0.207 |
| | 24 | 20.45 | 18.19 | 20.45 | 18.19 | 0.690 |
| | 52 | 19.31 | 17.05 | 29.54 | 21.73 | 0.343 |
| | 104 | 27.27 | 18.18 | 34.09 | 19.32 | 0.199 |
| PRTEE | 0 | 44.00 | 9.13 | 58.25 | 13.75 | **0.000** |
| | 2 | 24.75 | 12.00 | 35.00 | 17.63 | **0.017** |
| | 4 | 23.50 | 13.25 | 27.50 | 14.50 | 0.055 |
| | 8 | 19.75 | 13.75 | 26.75 | 17.00 | **0.048** |
| | 12 | 15.00 | 12.75 | 23.75 | 17.50 | **0.032** |
| | 24 | 8.00 | 13.25 | 24.00 | 17.88 | **0.000** |
| | 52 | 6.50 | 10.38 | 14.50 | 15.63 | **0.025** |
| | 104 | 2.50 | 6.50 | 10.00 | 14.75 | **0.010** |
| ΔPRTEE (vs week 0) | 2 | 14.75 | 11.00 | 14.50 | 14.00 | 0.715 |
| | 4 | 20.75 | 11.88 | 23.00 | 18.38 | 0.274 |
| | 8 | 24.25 | 14.50 | 27.50 | 14.75 | 0.324 |
| | 12 | 27.50 | 14.13 | 29.50 | 17.00 | 0.464 |
| | 24 | 35.50 | 17.25 | 28.00 | 18.75 | 0.354 |
| | 52 | 31.00 | 14.88 | 33.00 | 19.00 | 0.323 |
| | 104 | 37.50 | 14.00 | 40.00 | 16.25 | 0.186 |

Legend: QD, Quartile Deviation; PROMs, patient-reported outcome measures; VAS, Visual Analog Scale; QDASH, quick version of Disabilities of the Arm, Shoulder and Hand score; PRTEE, Patient-Rated Tennis Elbow Evaluation.
