# Supplementary material for: What Role Does PDGFA Gene Polymorphisms Play in Treating Tennis Elbow with PRP? A Prospective Cohort Study
Source: J Clin Med. 2022 Jun 17;11(12):3504. doi: 10.3390/jcm11123504 (PMC9224593; doi:10.3390/jcm11123504)
Supplement: Supplementary file 1 [file jcm-11-03504-s001.zip › Table S2.pdf]

Table S2. The frequencies (%) and medians (± QD) of basic demographic and clinical characteristics of patients in relation to genotypes of the *PDGFA* gene polymorphisms.

**Characteristics**	**rs1800814**			**rs2070958**			**rs62433334**		
	AA	AG	GG	CC	CT	TT	CC	CG	GG
n	37	60	35	23	56	53	65	57	10
Male sex, n (%)	12 (32.43)	31 (51.67)	12 (34.29)	8 (34.78)	30 (53.57) [5]	17 (32.08)	23 (35.38)	28 (49.12)	4 (40.00)
Age years, median ± QD	46.50 ± 4.00	45.00 ± 6.25	46.50 ± 5.50	45.00 ± 5.50	45.50 ± 6.00	47.00 ± 5.50	46.00 ± 6.50	46.50 ± 3.75	43.00 ± 5.00
BMI, median ± QD	25.47 ± 1.61	26.30 ± 3.35	25.08 ± 4.54	25.08 ± 2.66	26.48 ± 3.35	25.47 ± 1.73	25.88 ± 2.35	25.18 ± 2.13	25.08 ± 3.00
Cigarette smoking, n (%)	11 (29.73) [1]	6 (10.00)	5 (14.29)	4 (17.39)	6 (10.71)	12 (22.64)	9 (13.85)	13 (22.81)	0 (0.00)
Cigarettes n/day, median ± QD	0.00 ± 1.50	0.00 ± 0.00	0.00 ± 0.00	0.00 ± 0.00	0.00 ± 0.00	0.00 ± 0.00	0.00 ± 0.00	0.00 ± 0.00	0.00 ± 0.00
Alcohol units/ week, median ± QD	0.00 ± 1.00	1.00 ± 1.00	1.00 ± 2.00	0.00 ± 2.00	1.00 ± 2.00	1.00 ± 2.00	0.00 ± 1.00	1.00 ± 1.00	2.00 ± 1.50
Hypertension, n (%)	7 (18.92)	3 (5.00)	8 (22.86)	3 (13.04)	8 (14.29)	7 (13.21)	12 (18.46)	5 (8.77)	1 (10.00)
Follow up therapy									
Physical therapy, n (%)	17 (45.95)	19 (31.67) [2]	20 (57.14)	14 (60.87)	20 (35.71)	22 (41.51)	28 (43.08)	20 (35.09)	8 (80.00) [6]
Manual therapy, n (%)	8 (21.62)	18 (30.00)	11 (31.43)	14 (60.87) [3, 4]	18 (35.71)	11 (20.75)	18 (27.69)	17 (29.82)	2 (20.00)
NSAIDs, n (%)	8 (21.62)	22 (36.67)	11 (31.43)	8 (34.78)	19 (33.93)	15 (28.30)	19 (29.23)	20 (35.09)	2 (20.00)

Legend: BMI, body mass index; NSAIDs, nonsteroidal anti-inflammatory drugs; QD, quartile deviation.

[1] AA *vs* AG, *P* = 0.028

[2] AG *vs* GG, *P* = 0.015

[3] CC *vs* CT, *P* = 0.019

[4] CC *vs* TT, *P* = 0.000

[5] CT *vs* TT, *P* = 0.024

[6] GG vs CC+CG, *P* = 0.030
