# Supplementary material for: What Role Does PDGFA Gene Polymorphisms Play in Treating Tennis Elbow with PRP? A Prospective Cohort Study
Source: J Clin Med. 2022 Jun 17;11(12):3504. doi: 10.3390/jcm11123504 (PMC9224593; doi:10.3390/jcm11123504)
Supplement: Supplementary file 1 [file jcm-11-03504-s001.zip › Table S3.pdf]

Table S3. Whole blood (WB) and platelet-rich plasma (PRP) parameters values in individuals with particular genotypes of *PDGFA* gene polymorphisms (additive model).

Parameter	Source	Genotypes of rs1800814						*P* value			
		AA		AG		GG					
		**Median**	**±QD**	**Median**	**±QD**	**Median**	**±QD**	**Kruskal-Wallis test**	**AA vs AG**	**AA vs GG**	**AG vs GG**
PLT 10^9/l	WB	261.00	31.00	226.00	39.00	254.00	33.75	0.081			
PCT ml/l	WB	2.33	0.37	2.03	0.31	2.46	0.32	**0.044**	0.076	0.693	**0.039**
MPV fl	WB	9.00	0.55	9.40	0.80	9.10	0.75	0.913			
PDW fl	WB	16.00	0.15	16.10	0.25	16.05	0.15	0.581			
WBC 10^9/l	WB	6.92	1.53	5.98	0.95	6.24	1.01	0.080			
RBC 10^{12}/l	WB	4.56	0.30	4.72	0.33	4.69	0.24	0.290			
PLT 10^9/l	PRP	349.00	72.00	336.00	55.00	367.00	83.50	0.317			
PCT ml/l	PRP	0.29	0.07	0.29	0.06	0.31	0.07	0.351			
MPV fl	PRP	8.50	0.40	8.60	0.48	8.60	0.40	0.716			
PDW fl	PRP	14.60	0.20	14.60	0.25	14.50	0.20	0.815			
PDGF-AB ng/ml	PRP	8.74	11.90	7.16	2.06	9.77	3.90	**0.017**	0.328	0.686	**0.017**
**Parameter**	**Source**	**Genotypes of rs2070958**						***P* value**			
		**CC**		**CT**		**TT**					
		**Median**	**±QD**	**Median**	**±QD**	**Median**	**±QD**	**Kruskal-Wallis test**	**CC vs CT**	**CC vs TT**	**CT vs TT**
PLT 10^9/l	WB	246.00	34.50	229.00	31.50	246.00	48.75	0.235			
PCT ml/l	WB	2.46	0.33	2.05	0.35	2.31	0.29	0.062			
MPV fl	WB	9.40	0.90	9.10	0.75	9.00	0.65	0.418			
PDW fl	WB	16.00	0.20	16.10	0.15	16.05	0.15	0.770			
WBC 10^9/l	WB	6.24	1.04	6.11	1.05	6.77	1.24	0.467			
RBC 10^{12}/l	WB	4.67	0.13	4.77	0.29	4.56	0.32	0.071			
PLT 10^9/l	PRP	367.00	73.00	339.50	57.75	338.00	61.00	0.323			
PCT ml/l	PRP	0.33	0.07	0.30	0.05	0.28	0.06	0.244			
MPV fl	PRP	8.60	0.50	8.65	0.48	8.50	0.30	0.526			
PDW fl	PRP	14.50	0.20	14.60	0.23	14.60	0.20	0.548			
PDGF-AB ng/ml	PRP	9.31	3.41	8.05	2.84	8.27	1.68	0.133			

Parameter	Source	Genotypes of rs62433334						*P* value			
		CC		CG		GG					
		Median	±QD	Median	±QD	Median	±QD	Kruskal-Wallis test	CC vs CG	CC vs GG	CG vs GG
PLT 10^9/l	WB	238.00	32.00	246.00	50.50	227.00	30.50	0.769			
PCT ml/l	WB	2.19	0.43	2.33	0.33	2.31	0.19	0.445			
MPV fl	WB	9.00	0.70	9.40	0.85	8.90	1.20	0.186			
PDW fl	WB	16.00	0.15	16.10	0.20	16.10	0.20	0.519			
WBC 10^9/l	WB	6.61	1.17	6.28	0.97	4.89	1.45	0.189			
RBC 10^{12}/l	WB	4.66	0.23	4.69	0.42	4.77	0.33	0.550			
PLT 10^9/l	PRP	349.00	61.50	335.00	65.00	351.50	94.50	0.987			
PCT ml/l	PRP	0.31	0.05	0.30	0.05	0.29	0.08	0.892			
MPV fl	PRP	8.50	0.40	8.80	0.40	8.20	0.68	0.388			
PDW fl	PRP	14.50	0.20	14.60	0.25	14.50	0.45	0.485			
PDGF-AB ng/ml	PRP	8.30	1.72	8.70	2.91	7.67	5.70	0.930			

Legend: *MPV*, platelet volume; *PCT*, plateletcrit; *PDGFA*, platelet-derived growth factor alpha gene; *PDW*, platelet distribution width; *PLT*, platelets; *PRP*, platelet-rich plasma; *QD*, Quartile Deviation; *WB*, whole blood.
