# Supplementary material for: What Role Does PDGFA Gene Polymorphisms Play in Treating Tennis Elbow with PRP? A Prospective Cohort Study
Source: J Clin Med. 2022 Jun 17;11(12):3504. doi: 10.3390/jcm11123504 (PMC9224593; doi:10.3390/jcm11123504)
Supplement: Supplementary file 1 [file jcm-11-03504-s001.zip › Table S4.pdf]

Table S4. Whole blood (WB) and platelet-rich plasma (PRP) parameters values in individuals with particular variants of *PDGFA* gene polymorphisms (rs1800814, rs2070958 and rs62433334) in recessive/dominant model.

**Parameter**	**Source**	**rs1800814**				***P*, Mann-Whitney U test**
		**AA, AG**		**GG**		
		**Median**	**±QD**	**Median**	**±QD**	
PLT 10^9/l	WB	234.00	41.50	254.00	33.75	0.083
PCT ml/l	WB	2.18	0.33	2.46	0.32	**0.027**
MPV fl	WB	9.15	0.75	9.10	0.75	0.689
PDW fl	WB	16.10	0.15	16.05	0.15	0.677
WBC 10^9/l	WB	6.28	1.19	6.24	1.01	0.729
RBC 10^{12}/l	WB	4.65	0.31	4.69	0.24	0.597
PLT 10^9/l	PRP	339.00	57.50	367.00	83.50	0.131
PCT ml/l	PRP	0.29	0.06	0.31	0.07	0.169
MPV fl	PRP	8.60	0.40	8.60	0.40	0.787
PDW fl	PRP	14.60	0.25	14.50	0.20	0.826
PDGF-AB ng/ml	PRP	8.05	1.76	9.77	3.90	**0.019**
**Parameter**	**Source**	**rs2070958**				***P*, Mann-Whitney U test**
		**TT, CT**		**CC**		
		**Median**	**±QD**	**Median**	**±QD**	
PLT 10^9/l	WB	238.00	34.00	246.00	34.50	0.199
PCT ml/l	WB	2.19	0.34	2.46	0.33	**0.026**
MPV fl	WB	9.00	0.70	9.40	0.90	0.214
PDW fl	WB	16.10	0.15	16.00	0.20	0.576
WBC 10^9/l	WB	6.28	1.19	6.24	1.04	0.770
RBC 10^{12}/l	WB	4.68	0.31	4.67	0.13	0.726
PLT 10^9/l	PRP	339.00	58.50	367.00	73.00	0.206
PCT ml/l	PRP	0.29	0.06	0.33	0.07	0.167
MPV fl	PRP	8.60	0.40	8.60	0.50	0.591
PDW fl	PRP	14.60	0.20	14.50	0.20	0.679
PDGF-AB ng/ml	PRP	8.06	2.12	9.31	3.41	**0.047**
**Parameter**	**Source**	**rs62433334**				***P*, Mann-Whitney U test**
		**GG**		**CC, CG**		
		**Median**	**±QD**	**Median**	**±QD**	
PLT 10^9/l	WB	227.00	30.50	246.00	41.00	0.976
PCT ml/l	WB	2.31	0.19	2.31	0.37	0.925
MPV fl	WB	8.90	1.20	9.10	0.70	0.760
PDW fl	WB	16.10	0.20	16.10	0.15	0.678
WBC 10^9/l	WB	4.89	1.45	6.28	1.15	0.070
RBC 10^{12}/l	WB	4.77	0.33	4.66	0.30	0.493
PLT 10^9/l	PRP	351.50	94.50	342.50	63.25	0.972
PCT ml/l	PRP	0.29	0.08	0.30	0.06	0.867
MPV fl	PRP	8.20	0.68	8.60	0.40	0.296
PDW fl	PRP	14.50	0.45	14.60	0.23	0.857
PDGF-AB ng/ml	PRP	7.67	0.57	8.37	2.42	0.850

Legend: *MPV*, platelet volume; *PCT*, plateletcrit; *PDGFA*, platelet-derived growth factor alpha gene; *PDW*, platelet distribution width; *PLT*, platelets; *PRP*, platelet-rich plasma; *QD*, Quartile Deviation; *WB*, whole blood.
