# Supplementary material for: What Role Does PDGFA Gene Polymorphisms Play in Treating Tennis Elbow with PRP? A Prospective Cohort Study
Source: J Clin Med. 2022 Jun 17;11(12):3504. doi: 10.3390/jcm11123504 (PMC9224593; doi:10.3390/jcm11123504)
Supplement: Supplementary file 1 [file jcm-11-03504-s001.zip › Table S5.pdf]

Table S5. PROMs values in individuals with particular genotypes of *PDGFA* gene polymorphisms (rs1800814, rs2070958 and rs62433334) in additive model.

PROM	week	Genotypes of rs1800814						*P* value			
		AA		AG		GG					
		Median	±QD	Median	±QD	Median	±QD	Kruskal-Wallis test	AA vs AG	AA vs GG	AG vs GG
VAS	0	5.00	1.50	6.00	1.50	6.00	1.50	0.381			
	2	4.00	1.50	3.00	1.50	4.00	1.00	0.559			
	4	3.00	2.00	3.00	1.00	4.00	1.00	0.290			
	8	3.00	1.50	3.00	1.50	4.00	2.00	0.288			
	12	3.00	1.50	2.00	1.50	4.00	1.50	**0.039**	0.582	0.816	**0.038**
	24	3.00	2.00	1.50	2.50	3.00	1.50	0.331			
	52	1.00	2.50	1.00	2.00	2.50	1.50	0.421			
	104	1.00	1.50	1.00	1.00	1.00	1.50	0.668			
ΔVAS (vs week 0)	2	1.00	1.50	1.00	1.50	2.00	1.50	0.818			
	4	2.00	1.50	2.00	2.00	2.00	1.50	0.963			
	8	3.00	2.00	2.00	2.50	2.00	2.00	0.901			
	12	2.50	2.00	3.00	2.00	2.00	2.00	0.313			
	24	2.50	2.50	3.00	2.00	3.00	1.50	0.719			
	52	3.00	2.25	4.00	2.50	4.00	2.00	0.724			
	104	4.00	2.00	4.00	2.00	4.00	3.00	0.974			
QDASH	0	52.27	14.78	52.27	13.07	52.27	13.50	0.915			
	2	37.50	18.18	38.64	12.50	45.45	19.32	0.356			
	4	32.95	13.07	36.36	14.20	38.64	14.77	0.252			
	8	25.00	18.18	29.55	15.34	45.45	18.18	**0.049**	0.892	**0.047**	0.294
	12	25.00	18.18	27.27	13.07	43.18	19.32	0.060			
	24	29.55	22.73	19.32	23.86	27.27	20.45	0.425			
	52	18.18	23.86	13.64	21.59	26.14	20.45	0.276			
	104	9.09	20.45	13.64	21.59	15.91	14.77	0.548			
ΔQDASH (vs week 0)	2	6.82	18.53	6.81	13.07	0.00	12.50	0.200			
	4	11.36	18.18	16.59	15.91	9.09	14.77	0.191			
	8	14.77	21.01	19.32	19.37	6.82	19.32	0.078			
	12	18.18	19.87	22.73	14.38	4.54	20.46	**0.035**	1.000	0.125	**0.040**
	24	14.77	25.00	28.36	15.91	15.91	18.18	0.470			
	52	22.73	25.57	27.27	18.18	19.18	14.77	0.498			
	104	35.23	22.16	29.55	17.05	30.68	26.14	0.610			

PRTEE	0	52.50	17.50	51.00	13.88	52.00	10.00	0.668			
	2	29.25	15.00	27.50	16.38	37.00	16.25	0.509			
	4	19.00	14.13	24.75	11.88	29.50	15.50	0.208			
	8	19.50	13.50	21.75	12.25	33.00	19.75	0.119			
	12	18.00	13.75	16.00	10.25	34.00	16.75	0.071			
	24	17.00	17.00	10.00	19.75	23.50	15.00	0.493			
	52	12.00	15.75	9.50	12.50	16.50	17.00	0.624			
	104	3.50	12.50	6.50	12.75	9.50	11.25	0.678			
ΔPRTEE (vs week 0)	2	15.50	12.00	13.00	13.88	17.50	8.00	0.874			
	4	27.50	19.50	21.25	15.00	20.50	7.75	0.583			
	8	26.75	14.35	25.25	16.88	28.00	17.50	0.530			
	12	27.00	14.98	31.00	15.63	23.50	17.50	0.261			
	24	27.20	18.25	30.75	19.25	31.00	19.00	0.951			
	52	32.50	17.00	33.50	18.75	32.75	17.00	0.928			
	104	38.25	15.63	37.50	16.25	38.75	18.00	0.877			

**PROM**	**week**	**Genotypes of rs2070958**						***P* value**			
		**CC**		**CT**		**TT**					
		**Median**	**±QD**	**Median**	**±QD**	**Median**	**±QD**	**Kruskal-Wallis test**	**CC vs CT**	**CC vs TT**	**CT vs TT**
VAS	0	7.00	1.50	5.00	1.75	6.00	2.00	0.134			
	2	4.00	2.00	3.00	1.50	4.00	1.50	0.459			
	4	4.00	1.50	3.00	1.25	3.00	2.00	0.146			
	8	4.00	1.50	3.00	1.50	2.00	1.50	0.084			
	12	4.00	1.50	2.00	1.50	2.00	1.50	**0.028**	**0.030**	0.071	1,000
	24	3.00	2.00	2.00	2.00	3.00	2.00	0.293			
	52	2.00	1.00	1.00	1.50	1.00	2.50	0.751			
	104	1.00	1.50	1.00	1.00	1.00	1.50	0.762			
ΔVAS (vs week 0)	2	1.00	1.50	1.00	1.50	1.00	2.00	0.921			
	4	2.00	2.00	2.00	1.50	2.00	1.50	0.496			
	8	2.00	2.00	2.00	2.25	3.00	1.50	0.314			
	12	2.00	2.50	2.50	2.00	3.75	2.00	0.373			
	24	3.00	1.50	2.50	2.00	3.00	2.00	0.100			
	52	5.00	1.50	3.00	2.00	3.00	2.25	0.441			
	104	4.00	3.00	4.00	2.00	4.00	2.50	0.499			

QDASH	0	52.27	15.91	47.61	13.14	52.27	12.50	0.325			
	2	47.73	18.18	35.23	11.93	43.18	18.35	0.353			
	4	47.73	15.91	35.23	11.93	35.23	16.48	0.084			
	8	52.27	15.91	29.55	14.77	29.55	20.45	**0.014**	**0.026**	**0.018**	1.000
	12	43.18	18.18	27.27	13.07	25.00	20.45	**0.008**	**0.008**	**0.020**	1.000
	24	31.82	18.18	15.91	22.73	29.55	21.59	0.108			
	52	28.41	21.59	13.64	21.59	29.55	23.86	0.306			
	104	23.86	21.59	13.64	17.05	7.96	21.59	0.828			
ΔQDASH (vs week 0)	2	-0.01	9.18	6.81	13.70	9.09	17.39	0.197			
	4	4.54	13.63	14.77	15.34	15.90	18.18	**0.017**	0.123	**0.013**	0.836
	8	2.27	14.77	17.04	16.60	18.18	18.18	**0.012**	0.118	**0.009**	0.677
	12	0.00	12.50	22.72	14.21	23.64	19.20	**0.004**	**0.013**	**0.004**	1.000
	24	11.36	14.77	27.27	13.64	19.31	23.86	0.285			
	52	20.45	18.18	27.27	19.32	19.32	23.29	0.743			
	104	23.86	26.14	29.55	12.50	37.73	21.59	0.424			
PRTEE	0	54.00	10.00	50.75	11.75	52.50	16.00	0.643			
	2	39.50	15.75	26.25	16.50	29.25	15.00	0.545			
	4	36.00	16.25	26.00	12.00	21.00	14.25	0.112			
	8	33.00	17.25	21.75	16.00	19.50	13.50	**0.042**	0.105	**0.043**	1.000
	12	36.50	17.50	15.75	11.50	18.00	13.25	**0.007**	**0.007**	**0.017**	1.000
	24	24.50	14.75	10.00	19.50	15.00	16.75	0.293			
	52	19.25	16.00	9.50	14.00	15.00	15.50	0.607			
	104	9.25	14.75	7.25	11.50	3.25	14.00	0.912			
ΔPRTEE (vs week 0)	2	14.50	6.75	14.25	12.63	15.50	13.75	0.995			
	4	17.50	9.25	23.50	13.38	26.40	19.50	0.308			
	8	21.50	18.50	26.50	17.50	26.75	15.00	0.313			
	12	14.00	19.25	31.00	16.00	27.00	14.88	**0.050**	**0.046**	0.158	1.000
	24	31.00	19.25	31.25	16.25	27.20	18.25	0.769			
	52	33.25	15.25	38.00	20.00	27.75	15.38	0.662			
	104	38.00	18.50	38.25	16.25	37.50	16.00	0.957			

PROM	week	Genotypes of rs62433334						*P* value			
		CC		CG		GG					
		**Median**	**±QD**	**Median**	**±QD**	**Median**	**±QD**	**Kruskal-Wallis test**	**CC vs CG**	**CC vs GG**	**CG vs GG**
VAS	0	6.00	1.50	6.00	1.50	6.50	1.50	0.407			
	2	3.50	1.50	4.00	1.50	4.00	2.00	0.486			
	4	3.00	1.50	3.00	1.50	3.50	1.50	0.809			
	8	3.00	1.50	3.00	2.00	3.50	2.50	0.643			
	12	2.00	1.50	2.00	1.50	3.00	1.50	0.930			
	24	2.00	1.50	2.00	2.00	3.50	2.50	0.636			
	52	2.00	1.50	1.00	2.50	2.50	1.50	0.376			
	104	0.00	1.50	2.00	2.00	1.00	1.50	0.100			
ΔVAS (vs week 0)	2	1.00	1.50	1.00	1.50	1.00	1.50	0.833			
	4	2.00	2.00	2.00	1.50	2.50	1.00	0.555			
	8	2.00	2.00	3.00	2.00	3.00	1.50	0.717			
	12	2.00	2.25	3.00	2.00	4.00	2.00	0.423			
	24	2.50	2.00	3.00	2.00	4.00	1.00	0.913			
	52	4.00	2.50	2.00	2.50	5.00	1.50	0.182			
	104	4.00	2.50	3.00	2.50	5.00	1.00	0.166			
QDASH	0	52.27	14.77	52.27	11.37	55.68	12.50	0.548			
	2	37.50	14.20	43.18	15.91	37.50	26.14	0.705			
	4	35.23	14.20	36.36	12.50	43.18	17.05	0.686			
	8	31.82	19.32	34.09	18.18	40.91	17.05	0.694			
	12	27.27	15.91	34.09	17.05	30.68	21.59	0.942			
	24	29.55	21.59	20.45	21.59	25.00	22.73	0.766			
	52	13.64	22.73	22.73	22.73	15.91	10.23	0.372			
	104	13.64	20.45	19.09	21.59	2.27	4.55	0.095			
ΔQDASH (vs week 0)	2	6.82	12.50	4.54	12.50	-1.14	17.05	0.499			
	4	13.63	14.77	11.36	16.02	10.23	19.32	0.765			
	8	14.77	15.98	15.91	18.30	11.36	26.14	0.916			
	12	18.18	14.77	20.45	17.05	30.68	28.41	0.943			
	24	23.86	19.88	18.18	19.32	27.27	19.32	0.931			
	52	23.86	20.45	17.91	20.46	32.95	11.36	0.183			
	104	34.09	21.59	27.27	22.27	52.27	9.09	**0.031**	0.295	0.369	**0.044**
PRTEE	0	50.50	15.25	52.50	11.50	60.50	11.25	0.328			
	2	24.75	14.75	31.50	15.25	42.50	21.25	0.326			
	4	24.75	12.63	24.50	12.75	35.00	14.75	0.638			

	8	21.50	13.75	26.50	15.50	34.00	22.75	0.696			
	12	20.00	13.50	21.00	15.00	19.00	25.75	0.861			
	24	19.50	17.50	13.00	16.25	14.00	13.75	0.791			
	52	10.50	14.00	13.00	19.50	9.50	8.75	0.244			
	104	4.00	12.75	11.00	20.25	1.25	4.25	0.070			
ΔPRTEE (vs week 0)	2	15.00	11.25	14.50	13.25	14.75	8.00	0.992			
	4	18.00	13.00	26.40	10.75	28.75	14.50	0.734			
	8	24.75	14.38	28.00	16.75	21.50	22.00	0.782			
	12	27.50	17.00	30.50	12.75	37.00	23.50	0.379			
	24	27.75	16.25	31.00	19.00	37.50	19.00	0.393			
	52	36.50	18.00	29.00	17.25	38.50	10.25	0.141			
	104	38.50	15.75	33.50	15.50	55.25	11.25	**0.035**	0.650	0.189	**0.034**

Legend: *PDGFA*. platelet-derived growth factor alpha gene; QD. Quartile Deviation; PROMs. patient-reported outcome measures; VAS. Visual Analog Scale; QDASH. quick version of Disabilities of the Arm. Shoulder and Hand score; PRTEE. Patient-Rated Tennis Elbow Evaluation.
