# Supplementary material for: What Role Does PDGFA Gene Polymorphisms Play in Treating Tennis Elbow with PRP? A Prospective Cohort Study
Source: J Clin Med. 2022 Jun 17;11(12):3504. doi: 10.3390/jcm11123504 (PMC9224593; doi:10.3390/jcm11123504)
Supplement: Supplementary file 1 [file jcm-11-03504-s001.zip › Table S6.pdf]

Table S6. Platelets parameters and PROMs values in GG homozygotes and A allele carriers of the rs1800814 *PDGFA* gene polymorphism.

**PROMs**	week	**GG rs1800814** median	±QD	**AA+GA rs1800814** median	±QD	**p Mann-Whitney U test**
VAS	0	6.00	1.50	6.00	1.50	0.177
	2	4.00	1.00	4.00	1.50	0.406
	4	4.00	1.00	3.00	1.75	0.117
	8	4.00	2.00	3.00	1.50	0.117
	12	4.00	1.50	2.00	1.50	**0.029**
	24	3.00	1.50	2.00	2.50	0.210
	52	2.50	1.50	1.00	2.00	0.260
	104	1.00	1.50	1.00	1.50	0.435
ΔVAS (vs week 0)	2	2.00	1.50	1.00	1.50	0.648
	4	2.00	1.50	2.00	2.00	0.933
	8	2.00	2.00	3.00	2.25	0.750
	12	2.00	2.00	3.00	2.00	0.260
	24	3.00	1.50	3.00	2.00	0.935
	52	4.00	2.00	3.00	2.50	0.842
	104	4.00	3.00	4.00	2.00	0.938
QDASH	0	52.27	13.50	52.27	13.19	0.714
	2	45.45	19.32	38.64	14.20	0.171
	4	38.64	14.77	36.36	13.64	0.210
	8	45.45	18.18	29.55	17.05	**0.027**
	12	43.18	19.32	25.00	14.77	**0.018**
	24	27.27	20.45	25.00	22.73	0.193
	52	26.14	20.45	13.64	23.30	0.114
	104	15.91	14.78	11.36	21.59	0.394
ΔQDASH (vs week 0)	2	0.00	12.50	6.81	15.12	0.076
	4	9.09	14.77	15.45	15.91	0.112
	8	6.82	19.32	18.18	18.80	**0.029**
	12	4.54	20.46	22.72	15.68	**0.010**
	24	15.91	18.18	25.00	20.39	0.257
	52	19.18	14.77	27.27	19.45	0.250
	104	30.68	26.14	29.55	19.32	0.532
PRTEE	0	52.00	10.00	52.50	15.00	0.375
	2	37.00	16.25	28.00	15.00	0.268
	4	29.50	15.50	24.25	12.88	0.146
	8	33.00	19.75	20.00	12.75	**0.046**
	12	34.00	16.75	16.00	11.75	**0.024**
	24	23.50	15.00	13.00	17.50	0.236
	52	16.50	17.00	10.25	13.38	0.334
	104	9.50	11.25	6.25	12.75	0.442
ΔPRTEE (vs week 0)	2	17.50	8.00	14.00	14.00	0.646
	4	20.50	7.75	24.00	16.25	0.515
	8	28.00	17.50	25.50	15.75	0.279
	12	23.50	17.50	29.50	14.88	0.134
	24	31.00	19.00	29.50	18.75	0.823
	52	32.75	17.00	33.50	18.50	0.964
	104	38.75	18.75	37.50	16.25	0.870

Legend: *PDGFA*. platelet-derived growth factor alpha gene; QD. Quartile Deviation; WB. Whole Blood; PRP. Platelet-Rich Plasma; PROMs. patient-reported outcome measures; VAS. Visual Analog Scale; QDASH. quick version of Disabilities of the Arm. Shoulder and Hand score; PRTEE. Patient-Rated Tennis Elbow Evaluation.
