# Supplementary material for: What Role Does PDGFA Gene Polymorphisms Play in Treating Tennis Elbow with PRP? A Prospective Cohort Study
Source: J Clin Med. 2022 Jun 17;11(12):3504. doi: 10.3390/jcm11123504 (PMC9224593; doi:10.3390/jcm11123504)
Supplement: Supplementary file 1 [file jcm-11-03504-s001.zip › Table S7.pdf]

**Table S7.** Platelets parameters and PROMs values in CC homozygotes and T allele carriers of the rs2070958 *PDGFA* gene polymorphism.

| **Parameter** | | **CC rs2070958** | | **TT+TC rs2070958** | | **p Mann-Whitney U test** |
|---|---|---|---|---|---|---|
| | week | median | ±QD | median | ±QD | |
| VAS | 0 | 7.00 | 1.50 | 6.00 | 1.50 | 0.085 |
| | 2 | 4.00 | 2.00 | 4.00 | 1.50 | 0.326 |
| | 4 | 4.00 | 1.50 | 3.00 | 1.50 | 0.054 |
| | 8 | 4.00 | 1.50 | 3.00 | 1.50 | **0.032** |
| | 12 | 4.00 | 1.50 | 2.00 | 1.50 | **0.008** |
| | 24 | 3.00 | 2.00 | 2.00 | 2.00 | 0.161 |
| | 52 | 2.00 | 1.00 | 1.00 | 2.50 | 0.736 |
| | 104 | 1.00 | 1.50 | 1.00 | 1.50 | 0.478 |
| ΔVAS (vs week 0) | 2 | 1.00 | 1.50 | 1.00 | 1.50 | 0.865 |
| | 4 | 2.00 | 2.00 | 2.00 | 2.00 | 0.929 |
| | 8 | 2.00 | 2.00 | 3.00 | 2.00 | 0.642 |
| | 12 | 2.00 | 2.50 | 3.00 | 2.00 | 0.197 |
| | 24 | 3.00 | 1.50 | 3.00 | 2.00 | 0.987 |
| | 52 | 5.00 | 1.50 | 3.00 | 2.00 | 0.202 |
| | 104 | 4.00 | 3.00 | 4.00 | 2.25 | 0.644 |
| QDASH | 0 | 52.27 | 15.91 | 52.27 | 12.50 | 0.914 |
| | 2 | 47.73 | 18.18 | 38.64 | 14.77 | 0.190 |
| | 4 | 47.73 | 15.91 | 35.23 | 13.07 | **0.027** |
| | 8 | 52.27 | 15.91 | 29.55 | 17.05 | **0.004** |
| | 12 | 43.18 | 18.18 | 25.00 | 14.77 | **0.002** |
| | 24 | 31.82 | 18.18 | 25.00 | 21.59 | 0.090 |
| | 52 | 28.41 | 21.59 | 14.77 | 22.73 | 0.284 |
| | 104 | 23.86 | 21.59 | 15.91 | 21.59 | 0.546 |
| ΔQDASH (vs week 0) | 2 | -0.01 | 9.18 | 6.82 | 14.77 | 0.106 |
| | 4 | 4.54 | 13.63 | 15.90 | 15.91 | **0.008** |
| | 8 | 2.27 | 14.77 | 18.18 | 18.80 | **0.007** |
| | 12 | 0.00 | 12.50 | 22.72 | 16.88 | **0.001** |
| | 24 | 11.36 | 14.77 | 22.50 | 19.32 | 0.126 |
| | 52 | 20.45 | 18.18 | 22.73 | 21.59 | 0.513 |
| | 104 | 23.86 | 26.14 | 29.54 | 21.90 | 0.384 |
| PRTEE | 0 | 54.00 | 10.00 | 52.00 | 14.50 | 0.379 |
| | 2 | 39.50 | 15.75 | 28.00 | 15.00 | 0.273 |
| | 4 | 36.00 | 16.25 | 23.75 | 12.75 | **0.047** |
| | 8 | 33.00 | 17.25 | 20.00 | 15.00 | **0.014** |
| | 12 | 36.50 | 17.50 | 16.00 | 12.00 | **0.002** |
| | 24 | 24.50 | 14.75 | 13.50 | 17.25 | 0.147 |
| | 52 | 19.25 | 16.00 | 11.25 | 15.00 | 0.545 |
| | 104 | 9.25 | 14.75 | 9.00 | 12.75 | 0.685 |
| ΔPRTEE (vs week 0) | 2 | 14.50 | 6.75 | 15.00 | 13.00 | 0.925 |
| | 4 | 17.50 | 9.25 | 25.00 | 15.50 | 0.144 |
| | 8 | 21.50 | 18.50 | 26.50 | 16.13 | 0.155 |
| | 12 | 14.00 | 19.25 | 29.50 | 15.25 | **0.018** |
| | 24 | 31.00 | 19.25 | 29.50 | 18.63 | 0.965 |
| | 52 | 33.25 | 15.25 | 32.50 | 18.50 | 0.636 |
| | 104 | 38.00 | 18.50 | 38.00 | 16.50 | 0.947 |

Legend: *PDGFA*, platelet-derived growth factor alpha gene; QD, Quartile Deviation; WB, Whole Blood; PRP, Platelet-Rich Plasma; PROMs, patient-reported outcome measures; VAS, Visual Analog Scale; QDASH, quick version of Disabilities of the Arm, Shoulder and Hand score; PRTEE, Patient-Rated Tennis Elbow Evaluation.
