# Supplementary material for: What Role Does PDGFA Gene Polymorphisms Play in Treating Tennis Elbow with PRP? A Prospective Cohort Study
Source: J Clin Med. 2022 Jun 17;11(12):3504. doi: 10.3390/jcm11123504 (PMC9224593; doi:10.3390/jcm11123504)
Supplement: Supplementary file 1 [file jcm-11-03504-s001.zip › Table S8.pdf]

Table S8. Platelets parameters and PROMs values in GG homozygotes and C allele carriers of the rs62433334 *PDGFA* gene polymorphism.

Parameter		GG rs62433334		CC+CG rs62433334		p
	week	median	±QD	median	±QD	Mann-Whitney U test
VAS	0	6.50	1.50	6.00	1.50	0.213
	2	4.00	2.00	4.00	1.50	0.260
	4	3.50	1.50	3.00	1.50	0.576
	8	3.50	2.50	3.00	2.00	0.354
	12	3.00	1.50	2.00	1.50	0.709
	24	3.50	2.50	2.00	2.00	0.357
	52	2.50	1.50	1.50	2.50	0.792
	104	1.00	1.50	1.00	1.50	0.688
ΔVAS (vs week 0)	2	1.00	1.50	1.00	1.50	0.767
	4	2.50	1.00	2.00	2.00	0.520
	8	3.00	1.50	2.00	2.00	0.650
	12	4.00	2.00	3.00	2.00	0.423
	24	4.00	1.00	3.00	2.00	0.692
	52	5.00	1.50	3.00	2.00	0.122
	104	5.00	1.00	4.00	2.00	0.119
QDASH	0	55.68	12.50	52.27	13.64	0.510
	2	37.50	26.14	40.91	14.77	0.716
	4	43.18	17.05	36.36	13.64	0.388
	8	40.91	17.05	32.95	19.32	0.404
	12	30.68	21.59	28.41	17.05	0.790
	24	25.00	22.73	25.00	21.59	0.640
	52	15.91	10.23	18.18	23.30	0.843
	104	2.27	4.55	15.91	21.59	0.142
ΔQDASH (vs week 0)	2	-1.14	17.05	6.81	13.14	0.810
	4	10.23	19.32	12.50	15.34	0.875
	8	11.36	26.14	15.90	18.18	0.722
	12	30.68	28.41	18.18	15.91	0.735
	24	27.27	19.32	20.45	19.32	0.714
	52	32.95	11.36	20.45	21.59	0.271
	104	52.27	9.09	29.54	21.60	**0.040**
PRTEE	0	60.50	11.25	51.25	13.25	0.143
	2	42.50	21.25	28.00	14.50	0.227
	4	35.00	14.75	24.50	12.75	0.360
	8	34.00	22.75	22.00	14.50	0.399
	12	19.00	25.75	20.00	15.00	0.737
	24	14.00	13.75	15.25	17.50	0.689
	52	9.50	8.75	11.75	15.75	0.881
	104	1.25	4.25	7.75	14.00	0.211
ΔPRTEE (vs week 0)	2	14.75	8.00	14.75	12.25	0.906
	4	28.75	14.50	21.25	12.63	0.861
	8	21.50	22.00	25.50	15.75	0.842
	12	37.00	23.50	28.00	15.25	0.440
	24	37.50	19.00	30.50	17.75	0.259
	52	38.50	10.25	32.00	17.75	0.083
	104	55.25	11.25	37.50	16.25	**0.023**

Legend: *PDGFA*. platelet-derived growth factor alpha gene; QD. Quartile Deviation; WB. Whole Blood; PRP. Platelet-Rich Plasma; PROMs. patient-reported outcome measures; VAS. Visual Analog Scale; QDASH. quick version of Disabilities of the Arm. Shoulder and Hand score; PRTEE. Patient-Rated Tennis Elbow Evaluation.
